# The Pathoconnectivity Profile of Alzheimer’s Disease: A Morphometric Coalteration Network Analysis

**DOI:** 10.3389/fneur.2017.00739

**Published:** 2018-01-25

**Authors:** Jordi Manuello, Andrea Nani, Enrico Premi, Barbara Borroni, Tommaso Costa, Karina Tatu, Donato Liloia, Sergio Duca, Franco Cauda

**Affiliations:** ^1^GCS-fMRI, Department of Psychology, Koelliker Hospital, University of Turin, Turin, Italy; ^2^FOCUS Laboratory, Department of Psychology, University of Turin, Turin, Italy; ^3^Michael Trimble Neuropsychiatry Research Group, Birmingham and Solihull Mental Health NHS Foundation Trust, Birmingham, United Kingdom; ^4^Neurology Unit, Department of Clinical and Experimental Sciences, University of Brescia, Brescia, Italy

**Keywords:** brain alterations, coatrophy network, pathoconnectivity hubs, Alzheimer’s disease, tauopathy, gray matter atrophy, voxel-based morphometry

## Abstract

Gray matter alterations are typical features of brain disorders. However, they do not impact on the brain randomly. Indeed, it has been suggested that neuropathological processes can selectively affect certain assemblies of neurons, which typically are at the center of crucial functional networks. Because of their topological centrality, these areas form a *core set* that is more likely to be affected by neuropathological processes. In order to identify and study the pattern formed by brain alterations in patients’ with Alzheimer’s disease (AD), we devised an innovative meta-analytic method for analyzing voxel-based morphometry data. This methodology enabled us to discover that in AD gray matter alterations do not occur randomly across the brain but, on the contrary, follow identifiable patterns of distribution. This alteration pattern exhibits a network-like structure composed of coaltered areas that can be defined as *coatrophy network*. Within the *coatrophy network* of AD, we were able to further identify a core subnetwork of coaltered areas that includes the left hippocampus, left and right amygdalae, right parahippocampal gyrus, and right temporal inferior gyrus. In virtue of their network centrality, these brain areas can be thought of as *pathoconnectivity hubs*.

## Introduction

Widespread alterations of gray matter commonly characterize brain disorders. It has been suggested that neuropathological processes can selectively affect certain assemblies of neurons (1), which typically are at the center of crucial functional networks (1–7). Because of their topological centrality, these areas or network hubs form a *core set* that is more likely to be affected by neuropathological processes (1, 8–16). In particular, neurodegenerative diseases exhibit structural alterations that seem to distribute across the brain in network-like patterns (17, 18). These patterns, which we propose to call *morphometric coalteration networks* or, in the case of gray matter decreases, *coatrophy networks*, can be thought of as a form of pathological anatomical covariance (19, 20) and appear to develop according to the organization of brain connectivity (3, 4, 7). Studies aiming to investigate the networks formed by coaltered cerebral areas in the pathological brain are providing new insight for a better transdiagnostic and neurobiological understanding of the mechanisms at the root of brain disorders (21–23).

This is particularly true in the case of Alzheimer’s disease (AD). So far great efforts have been made in order to identify a prototypical pattern of gray matter atrophy due to AD, and to put it into correlation with clinical symptoms (24). It is now known that cortical thinning of specific brain sites can be already detected even before the appearance of the symptomatology and that the atrophy tends to increase when the condition worsens (25). Although the cortical reduction is commonly found in normal aging (26, 27), the pathological fingerprints of AD are mainly observed in a temporoparietal set of brain areas, including hippocampus, entorhinal cortex, precuneus, and posterior cingulate cortex (28, 29). The involvement of these regions has been repeatedly confirmed by meta-analytical studies, which have additionally found the alteration of the right superior frontal gyrus (30). According to Ferreira et al. (31) the left medial temporal lobe is the most impaired area in AD, even in the preclinical phases of the disease, so much so that the impairment of this area can be a good predictor of the clinical worsening of AD. A study of the relationship between the cortical thinning in AD and large-scale structural organization of the brain has revealed that AD reduces both the nodal centrality of temporal and parietal heteromodal association cortices and the positive correlation of thickness values normally found bilaterally between the parietal regions. In contrast, authors reported an increase of positive correlation among brain areas that are part of the default mode network (DMN) (32).

Recently, investigations into the cognitive deficits caused by AD have taken advantage of the methodology of network analysis (33, 34). According to this approach, altered brain areas can be represented by means of a set of nodes, linked together by means of edges representing different statistical values. Studies in this line of research have found that AD increases the correlation between the values of cortical thickness of the fusiform gyrus, temporal pole, parahippocampal gyrus, and cingulum, which are all in proximity to each other. Conversely, a decrease of the correlation has been observed between distant areas (35). Of note, it has been suggested that, by combining different sources of information: (i) large-scale structural networks data, (ii) values of cortical thickness, and (iii) the pace of cortical thinning along time, it could be possible to distinguish patients with AD from healthy controls with an accuracy of 96.1%, as well as predict the conversion of mild cognitive impairment (MCI) into AD 6 months before its clinical onset (36). These studies raise the issue of moving from group analysis to single-subject results, which is an essential aspect when dealing with potential biomarkers for diagnostic purposes and surrogate endpoints for disease-modifying clinical trials. Recent methods of single-subject graph measurements have allowed to link network alterations and cognitive decline. For instance, it has been showed that the more the network becomes disorganized, the worse the clinical condition is (37). Moreover, even in healthy subjects, it has been found an association between Aβ42 CSF low levels and alteration of network properties, which might be interpreted as a very early indication of an underlying pathological process (38). All these results provide evidence that the approach based on network analysis can bring valuable insight to clinical practice (33).

So far, at least four important mechanisms have been proposed to account for the distribution of brain alterations: transneuronal spread, nodal stress, shared vulnerability, and trophic failure (4, 5).

The first mechanism suggests that misfolded proteins (native peptides with an incomplete or incorrect folding, as well as *de novo* polypeptides that become prone to self-aggregation) can diffuse along neuronal pathways (18, 39–41). Increasing evidence indicate that the spread of misfolded proteins presents several similarities to the plasma membrane prion protein intercellular transfer, along axonal fibers, potentially contributing to disease progression (42). This mechanism has been demonstrated in neurodegenerative diseases, such as Alzheimer’s, Parkinson’s, Huntington’s, amyotrophic lateral sclerosis, and tauopathies (43, 44); more recently it has been also generalized to other brain disorders (45).

The second mechanism hypothesizes that the functional stress of the network hubs may result in a greater vulnerability of these areas (1, 4, 14, 46). This susceptibility has been supported in human beings with *in vivo* neuroimaging techniques and voxel-based meta-analyses (14).

The third mechanism suggests that certain brain regions sharing gene or protein expressions may be more vulnerable to neuropathology (4, 47–51), with a potential relationship between gene expressions and connectivity patterns (51, 52).

Finally, the fourth mechanism hypothesizes a disruption in the production of trophic factors, which could bring about the deterioration of neural wiring (4, 5, 53, 54).

If we consider the case of AD, neuropathological signatures, namely amyloid-β (Aβ) plaques and neurofibrillary tangles, are already present in the preclinical phase of the disease, with further spreading during progression. In fact several years before the clinical onset of AD, Aβ, and tau progressively accumulate in the brain with a certain degree of spatial specificity as well as a partial overlap among the two deposits (55). The relationship between tau and amyloid deposits in the cerebral cortex seems to have a hierarchical organization, with tau and Aβ clusters exhibiting distinctive intramodal and intermodal characterizations (56). These findings would support the view of AD as an amyloid-facilitated tauopathy (57). Furthermore, Aβ and tau propagation and the subsequent deposition and cytotoxicity effects appear to occur mainly between anatomically interconnected areas, thus affecting the functional communication among them (58).

The concept of a gradual spread of pathological signs is a crucial aspect put forward by recent theoretical models. Raj et al. (3) have proposed a network diffusion model of disease progression in dementia, according to which the propagation of pathogenic proteins follows the regional concentration gradients under the spatial constraints defined by brain connectivity. Other authors have proposed a stochastic epidemic spreading model to describe intra-brain Aβ propagation and deposition processes, according to which regions with a higher connectivity degree are the main target of Aβ, thus suggesting that brain hubs are the more exposed to the negative effects of these aberrant proteins (40). Finally, in addition to focusing on misfolded proteins and propagation pathways, a further interesting approach suggests the need to investigate the relationship between these two factors (18). This model considers molecular nexopathies as conjunctions of pathogenic protein and brain networks. Key factors are therefore supposed to be structural/functional developmental factors and differential vulnerability of neural connections. Accordingly, long-range axonal connections may be more vulnerable to Aβ, so that functional and structural alterations could occur within the large-scale distributed frontotemporoparietal network, such as the one that supports the DMN processing.

In order to identify and study the coatrophy network of AD, we devised an innovative meta-analytic method for analyzing voxel-based morphometry (VBM) data. This methodology enabled us to address the following issues:

- How do gray matter alterations distribute across the brain affected by AD?
- Is it possible to recognize a network-like structure in the pattern formed by these coaltered areas?
- Can specific clusters of coaltered areas be identified within the coatrophy network of AD?

## Materials and Methods

### Selection of Studies

On March 2017, we performed with the software Sleuth an extensive meta-analytic search in the BrainMap VBM database (www.brainmap.org) (59–61). All the studies that fulfilled the following criteria were retrieved: “Contrast is Gray Matter”; “Context is Disease Effect”; “Observed Changes is Controls > Patients” and “Diagnosis is Alzheimer’s Disease.” Results were controlled so as to keep only experiments comparing subjects diagnosed with AD against healthy controls. Our search focused on gray matter decreased values only, as the development of AD is strongly characterized by axonal deterioration and neuronal loss that result in brain atrophy (62). Furthermore, thus far just a few VBM studies have investigated gray matter increase in AD, so that these data were not sufficient for obtaining reliable results with our meta-analytical methods.

To ensure a transparent description of the selection process, we followed the “PRISMA Statement” international guidelines (63, 64) (Figure S1 in Supplementary Material). The characteristics of the sample can be viewed in Table 1.

**Table 1:** Selected studies for the meta-analysis.

ID	Reference	Journal	AD patients			Age			Scanner field (T)	Slice thick (mm)	Smoothing (mm)	Software
			Men	Women	Total	Min	Max	Mean ± SD				
1	Agosta et al. (65)	Radiology	14	9	23	–	–	74.6 ± 8.6	1.5	0.9 × 0.5 × 0.5	8	SPM5
2	Baron et al. (66)	NeuroImage	8	11	19	63	85	73 ± 5	1.5	1.5 × 1 × 1	12	SPM2
3	Baxter et al. (67)	Journal of Alzheimer’s Disease	11	4	15	64	91	75.5 ± 7.8	1.5	1.5 × 0.9 × 0.9	12	SPM2
4	Berlingeri et al. (68)	Behavioral Neuroscience	8	13	21	–	–	76.5	1.5	1 × 1 × 1	–	SPM2
5	Boxer et al. (69)	Archives of Neurology	8	3	11	–	–	69.6 ± 8.2	1.5	–	12	SPM99
6	Bozzali et al. (70)	Neurology	11	11	22	–	–	67.9 ± 7.6	1.5	1	12	SPM2
7	Brenneis et al. (71)	NeuroReport	3	7	10	–	–	73.1 ± 7.6	1.5	1 × 1 × 1	–	SPM99
8	Canu et al. (72)	Neurobiology of Aging	13	29	42	–	–	77.8 ± 4.8	1	1.3	8	SPM8
								62.5 ± 4.5				
9	Chetelat et al. (73)	NeuroReport	7	9	16	63	85	72.1 ± 5.8	1.5	2 × 2 × 2	12	SPM99
10	Farrow et al. (74)	Psychiatry Research NeuroImaging	–	–	14	68	87	77 ± 7	1.5	1 × 1 × 1	8	SPM2
								78 ± 7				
11	Feldmann et al. (75)	Psychiatry Research	4	2	6	–	–	61.1 ± 7.7	1	0.8	8	SPM2
12	Frisoni et al. (76)	Journal of Neurology, Neurosurgery, and Psychiatry	6	23	29	53	86	74 ± 9	1.5	1.3	8	SPM99
13	Guo et al. (77)	Neuroscience Letters	6	7	13	58	81	72.1 ± 6.5	3	0.5 × 0.5 × 1	8	SPM2
14	Hall et al. (78)	Alzheimers Dementia	16	31	47	–	–	83.2 ± 5	1.5	1 × 1 × 1	10	SPM2
								79.4				
15	Hamalainen et al. (79)	Neurobiology of Aging	5	10	15	62	83	73.1 ± 6.7	1.5	2 × 2 × 2	–	SPM2
16	Hirao et al. (80)	Nuclear Medicine Communications	32	29	61	48	87	70.6 ± 8.4	1.5	1.23	12	SPM2
17	Honea et al. (81)	Alzheimer’s Disease and Related Disorders	23	37	60	–	–	74.3 ± 6.3	3	1 × 1 × 1	10	SPM5
18	Ishii et al. (82)	European Journal of Nuclear Medicine and Molecular Imaging	8	22	30	–	–	66.8 ± 7.0	1.5	1.5	12	SPM99
19	Kanda et al. (83)	European Journal of Nuclear Medicine and Molecular Imaging	–	–	20	–	–	65	1.5	1.5	–	SPM2
20	Kawachi et al. (84)	European Journal of Nuclear Medicine and Molecular Imaging	9	23	32	–	–	67 ± 4.5	1.5	–	12	SPM99
21	Kim et al. (85)	Journal of Clinical Neuroscience	–	–	61	–	–	70.1 ± 5.0	3	1	12	SPM2
								71.1 ± 6.1				
								73.9 ± 5.5				
22	Matsuda et al. (86)	Journal of Nuclear Medicine	11	4	15	59	81	71.1 ± 7.1	1	1.23	12	SPM99
23	Matsunari et al. (87)	Journal of Nuclear Medicine	12	15	27	–	–	68.6 ± 6.8	1.5	0.78 × 1.04 × 1.4	12	SPM2
24	Mazere et al. (88)	NeuroImage	3	5	8	–	–	80 ± 6.8	1.5	1	8	SPM2
25	Miettinen et al. (89)	European Journal of Neuroscience	5	11	16	63	83	74.8 ± 5.4	1.5	2 × 2 × 2	12	SPM2
26	Ohnishi et al. (90)	American Journal of Neuroradiology	11	15	26	59	79	72.1 ± 1.1	1.5	–	12	–
27	Rabinovici et al. (91)	American Journal of Alzheimer’s Disease and Other Dementias	5	6	11	–	–	64.5 ± 9.7	1.5	–	12	SPM2
28	Rami et al. (92)	International Journal of Geriatric Psychiatry	9	22	31	–	–	76.4 ± 6.8	1.5	1.5	10	SPM2
29	Remy et al. (93)	NeuroImage	1	7	8	–	–	72.2 ± 10.8	1.5	1 × 1 × 1	8	SPM2
30	Shiino et al. (94)	NeuroImage	19	21	40	55	82	71.1 + 9.7	1.5	–	12	SPM99
31	Takahashi et al. (95)	American Journal of Neuroradiology	20	31	51	–	–	72.6 ± 2.9	1.5	1.5	6	SPM8
32	Testa et al. (96)	Journal of Magnetic Resonance Imaging	2	5	7	–	–	73 ± 11	1.5	2 × 2 × 2	8	SPM99
33	Waragai et al. (97)	Journal of the Neurological Sciences	7	8	15	–	–	71 ± 5.1	1.5	2	12	SPM5
34	Whitwell et al. (98)	Neurobiology of Aging	16	22	38	–	–	65.3 ± 6.9	1.5	1.6	8	SPM2
35	Xie et al. (99)	Neurology	8	5	13	62	82	71.7	1.5	1.6	8	SPM2
36	Zahn et al. (100)	Psychiatry Research NeuroImaging	4	6	10	–	–	66.5 ± 8.9	1.5	1.5 × 1.5 × 1.5	8	SPM2

*Where no information about slice thickness was provided, the voxel-size was expressed. The items are the result of the entire selection process as shown in PRISMA (Figure S1 in Supplementary Material) flow chart*.

### Anatomical Likelihood Estimation (ALE) and the Creation of Modeled Activation (MA) Maps

Voxel-based morphometry data were statistically elaborated with the procedure of the ALE. ALE is a voxel-based meta-analytical technique that models the spatial coherence of different results (101–103). A three-dimensional Gaussian probability distribution is then centered on each focus of every experiment, using the following formula:

$$p(d)=\frac{1}{\sigma^{3}\sqrt{{\left(2\pi \right)}^{3}}}{\text{e}}^{-\frac{d^{2}}{2\sigma^{2}}},$$

in which *d* refers to the Euclidean distance between voxels and the considered focus, while e refers to the spatial uncertainty. The SD can be obtained by means of the full-width half-maximum, such as:

$$\sigma =\frac{\text{FWHM}}{\sqrt{8\text{ln}2}}.$$

The combination of these Gaussian distributions constructs a MA map for each experiment. The definite ALE map is finally generated by uniting the MA maps. ALE maps were thresholded at a voxel-level FWD *p* < 0.05, in line with Eickhoff et al. (102, 104, 105). Given a specific threshold for cluster forming, a null distribution of cluster sizes was derived by simulating a long series of experiments with the same characteristics of real data and then by generating an ALE map. The score histogram so obtained was eventually employed to assign a threshold *p*-value.

### Construction of the Morphometric Coatrophy Network

To identify the distribution of gray matter alterations, we have developed a novel methodology capable of constructing the morphometric coalteration networks associated with brain disorders. Our analysis can in fact discover whether an altered brain area, say A, is statistically related to the alteration of one or more other brain areas (B, C, etc.). Thus, our analysis can construct the morphometric coatrophy network composed of the areas occurring to be altered together and, subsequently, investigate within the coatrophy network (i) how an altered region is statistically associated with other altered regions and (ii) which regions are likely to be involved in a more widespread net of alterations.

### Node Creation and Labeling

We superimposed the ALE map on the Talairach atlas so as to distinguish automatically the anatomical regions identified by the ALE algorithm. If (at least) 20 voxels of the ALE map were found to be inside a certain area of the atlas, then this area was considered to be altered. We chose this cluster threshold so that less relevant regions could be excluded. We employed a peak detection algorithm to identify the local maxima of the ALE map, and we subsequently selected only those peaks that were greater than the 90 percentile of the value distribution. This set was further reduced by applying a minimum interpeak distance of 10 mm. Finally, we positioned a node, labeled on the basis of the Talairach atlas, in correspondence of every survived peak.

### Thresholding Values Applied during Nodes Creation and Their Rationale

As described in the previous paragraph, three thresholds were applied during the nodes creation procedure.

The first threshold regulates the minimum number of voxel (i.e., 20 voxels) necessary to consider a brain area as altered. The rationale behind this threshold is to exclude from the coatrophy network nodes representing minimally (or, from a meta-analytical point of view, rarely) altered brain areas, thus improving and simplifying the interpretability of the results without losing highly relevant information. However, even considering brain areas in which only one voxel is altered, the results would have not been spurious, since ALE maps were voxel-level thresholded, which implies that each single voxel contains statistically significant information (104) (see Figure S2 in Supplementary Material for the visualization of the network obtained with different threshold values). This choice, however, would have unnecessarily increased the complexity of the coatrophy network.

The second threshold, applied to the peaks-value distribution, allowed us to include in the network only nodes representing those areas for which there is a very high consensus between different experiments (i.e., high ALE value) (104). Even in this case, this threshold could have been removed; all the nodes that can be created with the present methodology represent statistically significant effects, since they can only lie inside the anatomical regions identified by the ALE algorithm, which already has its own statistical thresholding step (see Figure S3 in Supplementary Material for the visualization of the network obtained with different threshold values).

Finally, the interpeaks distance was chosen considering the mean value (10.2 mm; SD = 0.4 mm) of uncertainty in spatial location associated with the reported coordinate discussed in Eickhoff et al. (101).

Therefore, the only effect of those thresholds on our data is to decrease the redundancy of the network, so as to obtain clearer results to be visualized and further analyzed, minimizing the information loss.

### Coatrophy Distribution

From the set of the nodes as defined in the previous paragraph, we constructed a *N* × *M* matrix or a coalteration matrix, in which each row referred to an experiment, whereas each column referred to a network node. On the basis of a Bernoulli generation data model, we constructed a probability distribution of joint alteration values for each pair of nodes. In other words, for any couple of nodes (*a* and *b*), we were able to describe their four conjoint states of alteration by means of two binary variables: (1) *a* and *b* both altered; (2) *a* and *b* both unaltered; (3) *a* altered and *b* unaltered; and (4) *a* unaltered and *b* altered. Consequently, the following four probabilities were obtained by the frequencies of the different combinations of all experiments:

$$\theta_{1}=P\left(a=1,b=1\right),$$

$$\theta_{2}=P\left(a=1,b=0\right),$$

$$\theta_{3}=P\left(a=0,b=1\right),$$

$$\theta_{4}=P\left(a=0,b=0\right).$$

These formulas refer to the conjoint frequencies of a couple of nodes (*a* and *b*) in all their four possible combinations. Table 2 shows the marginal probabilities for each couple of nodes.

**Table 2:** Marginal probabilities between altered and unaltered nodes.

	Node*a*			
	Node b	Altered	Unaltered	
	**Altered**	θ_1_	θ_3_	θ_1_ + θ_3_
	**Unaltered**	θ_2_	θ_4_	θ_2_ + θ_4_
		θ_1_ + θ_2_	θ_3_ + θ_4_	1

On the grounds of these four probabilities, we have applied the Patel’s *k* index (106)—which has been validated with simulated data by Smith et al. (107)—in order to calculate the degree of coalteration between nodes. This index can measure the probability that two nodes (*a* and *b*) are actually coaltered against the probability that node *a* and node *b* are altered independently of each other. Patel’s *k* is calculated as follows:

$$\kappa =\left(\vartheta_{1}-E\right)/\left(D\left(\text{max}\left(\vartheta_{1}\right)-E\right)+\left(1-D\right)\left(E-\text{min}\left(\vartheta_{1}\right)\right)\right),$$

where

$$E=\left(\vartheta_{1}+\vartheta_{2}\right)\left(\vartheta_{1}+\vartheta_{3}\right),$$

$$\text{max}\left(\vartheta_{1}\right)=\text{min}\left(\vartheta_{1}+\vartheta_{2},\vartheta_{1}+\vartheta_{3}\right),$$

$$\text{min}\left(\vartheta_{1}\right)=\text{max}\left(0,2\vartheta_{1}+\vartheta_{2}+\vartheta_{3}-1\right).$$

The numerator refers to the difference between the probability that *a* and *b* are altered together and the expected probability that *a* and *b* are altered independently of each other. The denominator refers to a weighted normalizing constant. Min (ϑ_1_) refers to the maximum value of the conjoint probability *P*(*a,b*), given *P*(*a*) and *P*(*b*), whereas max (ϑ_1_) refers to the minimum value of *P*(*a,b*), given *P*(*a*) and *P*(*b*). Patel’s *k* index has values that range from −1 to 1. A value of |*k*| that is close to 1 indicates a high degree of connectivity between nodes. The statistical significance of this index was assessed with a Monte Carlo algorithm that simulated a multinomial, generative model, which took into consideration the alteration of all nodes. This statistical procedure obtained an estimation of *p*(*k*|*z*) by sampling a Dirichlet distribution and by calculating the samples’ amount for which *k* > *e*, where *e* was the threshold of statistical significance set at *p* < 0.01.

### Topological Analysis

We defined our system of interconnected nodes as a network of coatrophy areas and examined it with the network analyzer included in Cytoscape 3.5.1 (108, 109). We were therefore able to achieve a good and reliable description of the net formed by the coatrophy areas under both the aspects of brain structure and functional organization.

### Node Degree and Edge Betweenness

The node degree was defined as the number of edges linked to a node. We employed this parameter in order to detect the nodes that were more connected within the network, which are commonly considered as brain hubs. In turn, the parameter of edge betweenness was defined as the number of the shortest routes that go through an edge in a graph or a network (110). Thus, edges exhibiting high values of betweenness are supposed to be involved in a large number of shortest routes, so that their elimination is likely to have an impact on communication between many couples of nodes.

### Network Clustering

Given the great number of nodes as well as the high density of edges within the coatrophy network, we used the *k*-core decomposition algorithm (111, 112)–as it is implemented in the clusterMaker plugin for Cytoscape—to detect a central subnetwork of highly interconnected nodes. This algorithm eliminates all the nodes showing a degree that is lesser than a user-defined *k*, thus deriving from the original network the highest connected subgraph.

## Results

### Common Patterns of Morphometric Alterations

**Figure 1:**
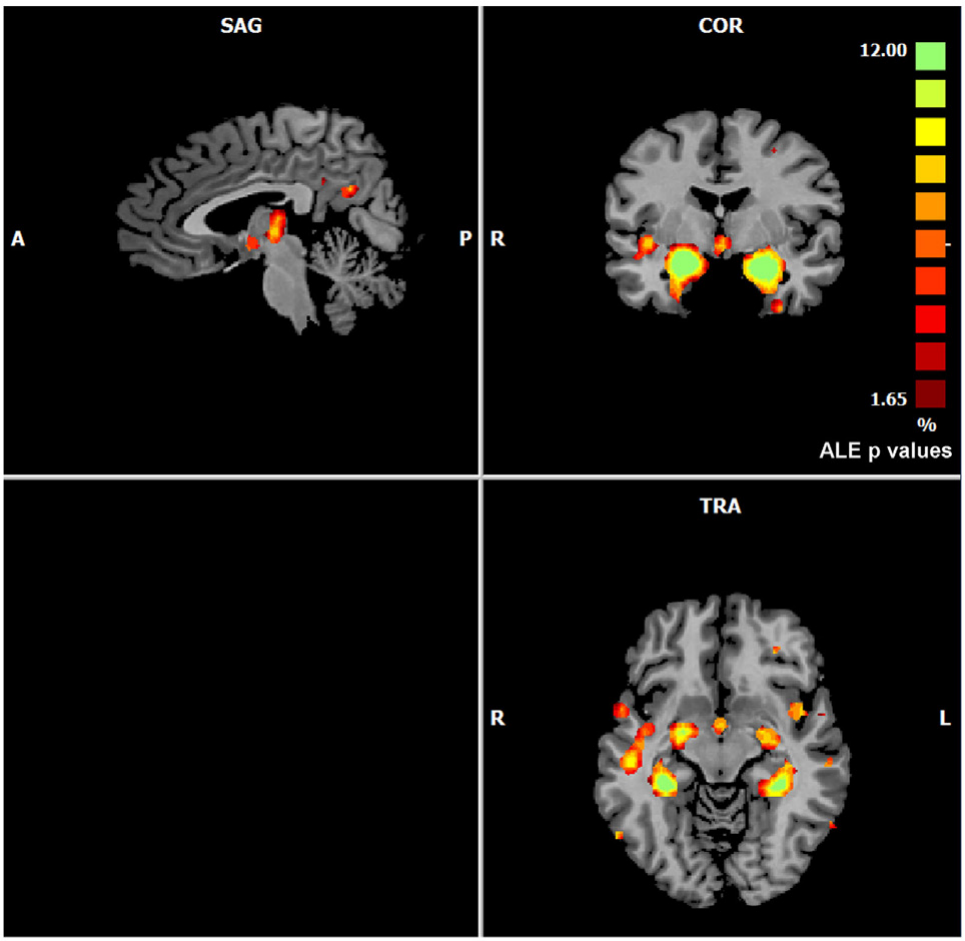
Gray matter anatomical likelihood estimation (ALE) results. The image summarizes the results of all the experiments considered in this meta-analysis. Colors from red to green show gray matter decreases [ALE maps were thresholded using voxel-level FWD *p* < 0.05 (104) and visualized using Brainvoyager QX].

The ALE performed on all the data retrieved by our search (57 experiments, 883 subjects, and 691 foci) showed that gray matter alterations caused by AD are mainly located in the right medial frontal gyrus, the right inferior frontal gyrus, the left inferior parietal lobule, the right midcingulate gyrus, the left supramarginal gyrus, the right angular gyrus, the bilateral fusiform gyrus, the right precuneus, the bilateral insula, the right thalamus, the bilateral superior temporal gyrus, the bilateral superior temporal pole, the bilateral hippocampus, the bilateral parahippocampal gyrus, the bilateral amygdala, and the left caudate nucleus (Figure 1).

### Morphometric Coatrophy Network

**Figure 2:**
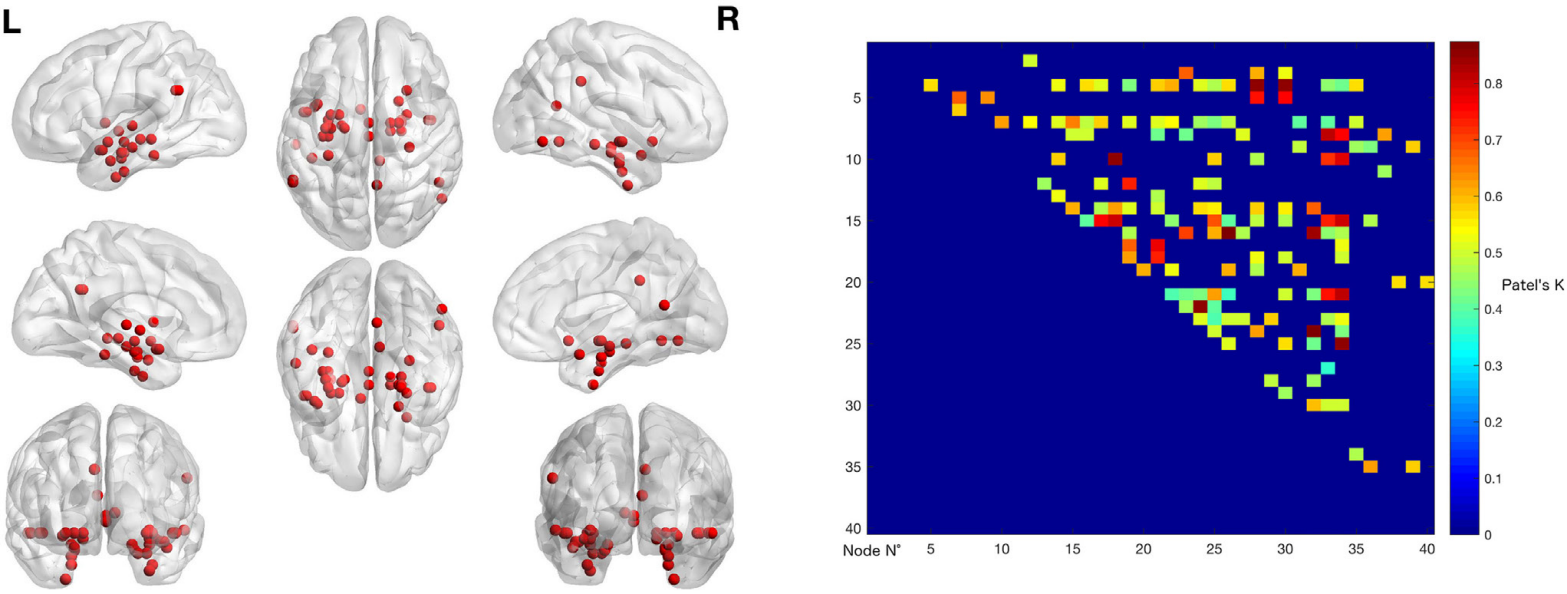
The left panels shows the nodes that entered the coatrophy calculation. The right panel shows the coatrophy matrix. Colors from blue to red indicate increasing Patel’s *k* values (i.e., increasing coalteration probabilities).

**Figure 3:**
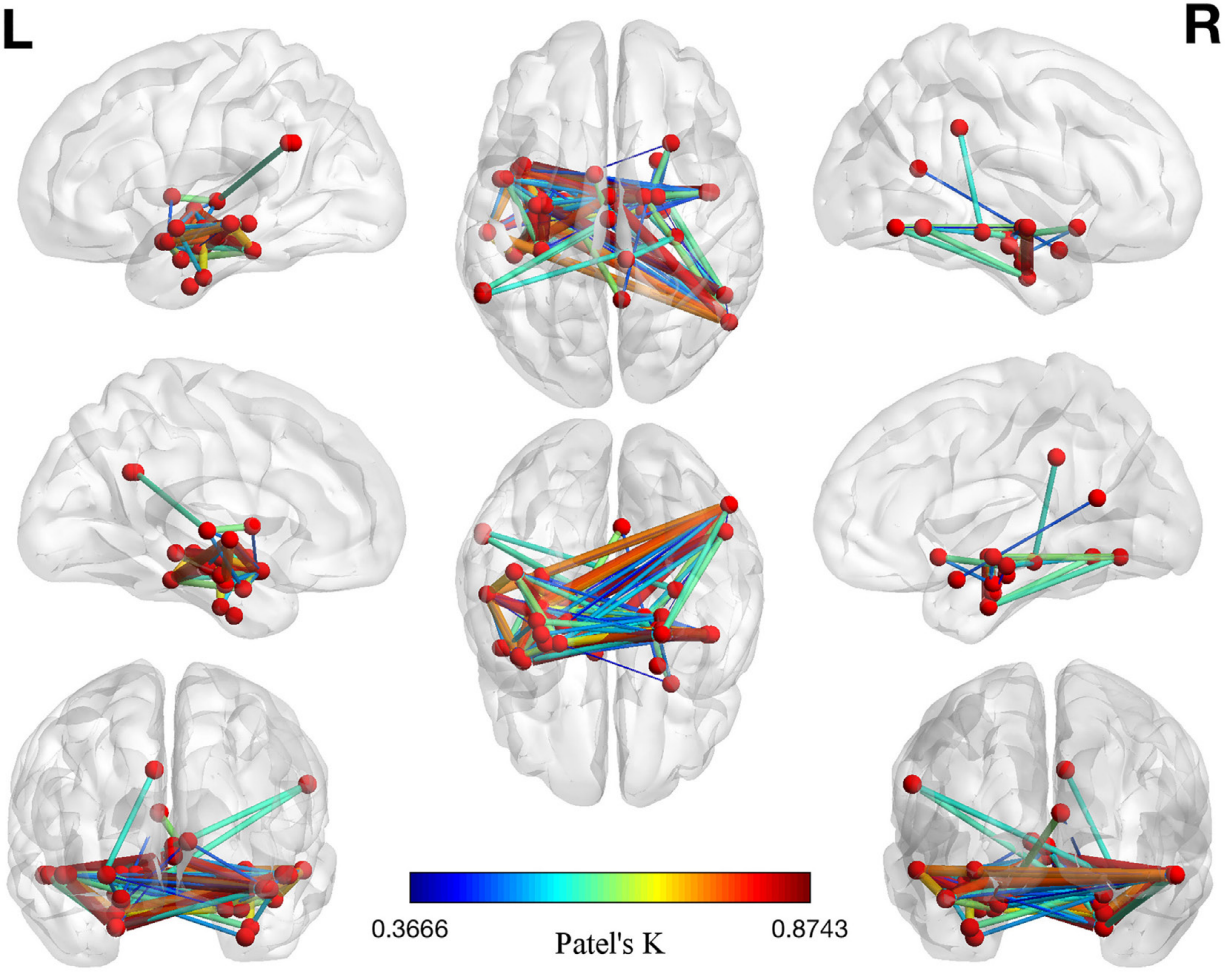
Morphometric coatrophy network results. Colors from blue to red indicate increasing Patel’s *k* values (i.e., increasing coalteration probabilities).

The left panel of Figure 2 illustrates the 40 nodes used to build the coatrophy network, while the heat map in Figure 2 shows the relationship between the elements of each possible couple of nodes measured by Patel’s *k* index. Figure 3 illustrates the whole coatrophy network: the colors’ scale ranges from blue to red for the 146 edges and indicates an increase in *k* values. Edges are to be assumed as undirected.

Many nodes densely interconnected characterize the temporal lobe, especially the hippocampus and the parahippocampal gyrus. In contrast, only one node characterizes other brain areas, such as the cingulate cortex and precuneus. Although all the edges that are shown are statistically significant, the ones with the highest *k* value are those involving the left hippocampus, bilateral amygdala, right parahippocampal gyrus, and right inferior temporal lobe (Tables S1 and S2 in Supplementary Material).

**Figure 4:**
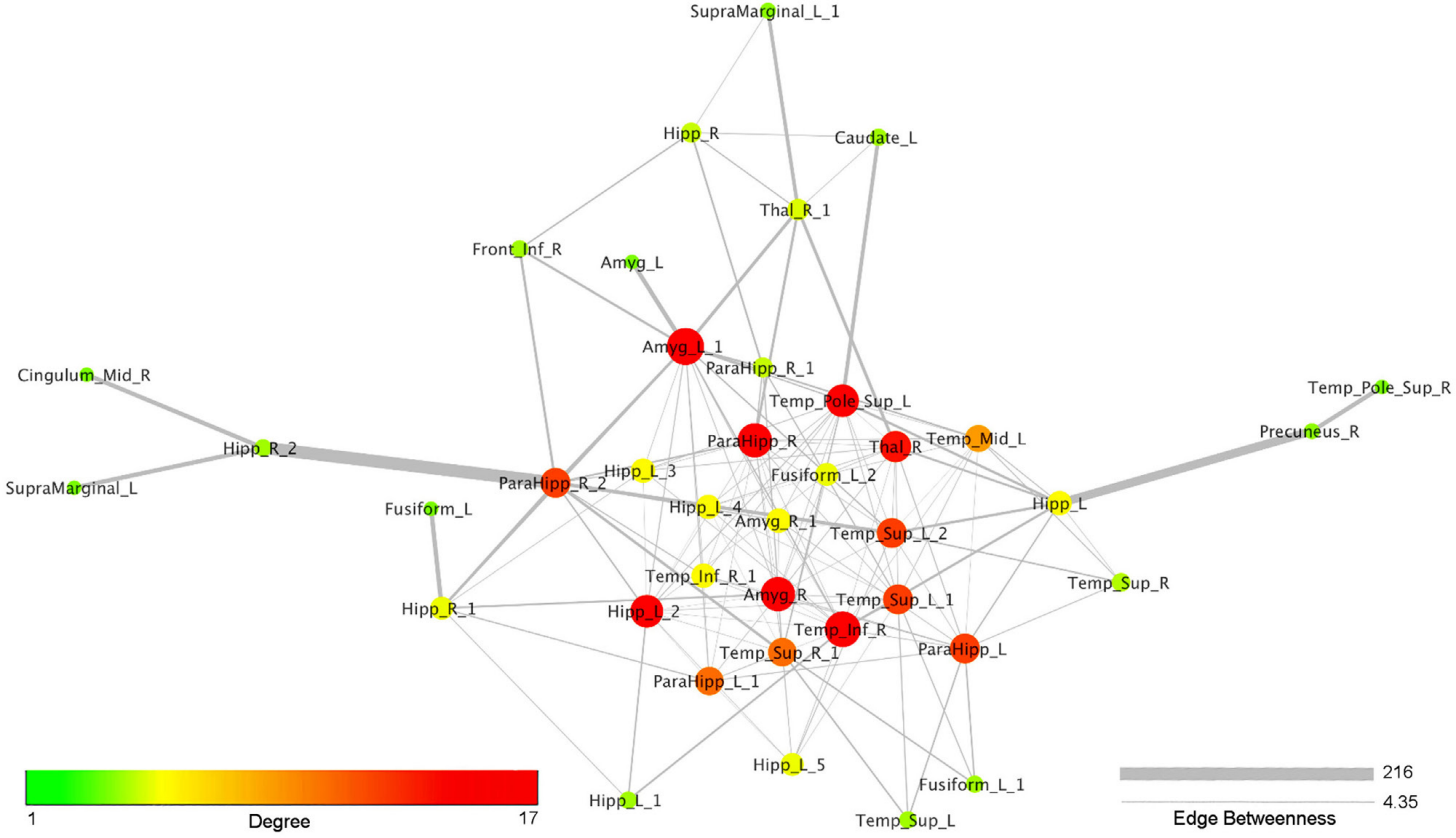
Topological analysis of the coatrophy network of Alzheimer’s disease (organic yFiles Layout). Colors and dimensions of nodes indicate their topological degree (smaller node = lower degree; from green to red = from lower to higher values). Thickness of edges indicate the degree of edge betweenness (smaller edge = lower degree).

Figure 4 reports the organic option of the yFiles Layouts available in Cytoscape 3.5.1 (based on a spring-embedded algorithm) attributed to the coatrophy network. Thick links connect the nodes located in the temporal cortex, parahippocampal gyrus, amygdala, and thalamus. The right precuneus is connected to the rest of the network just through one edge projecting to the left hippocampus, whereas the right cingulate cortex is connected to the network core through the right hippocampus and the right parahippocampal gyrus. In Figure 4, colors and dimensions of nodes are proportional to their network degree values. In particular, Amyg_L_1 shows the highest degree value (17), followed by Temp_Inf_R (16). In turn, Fusiform_L, Amyg_L, Temp_Pole_Sup_R, SupraMarginal_L, and Cingulum_Mid_R exhibit the lowest degree value (1). The edges’ thickness is proportional to their degree of edge betweenness. The edge linking the nodes Hipp_R_2 and ParaHipp_R_2 shows the highest value, while the edge between Amyg_R and ParaHipp_L_1 shows the lowest one.

**Figure 5:**
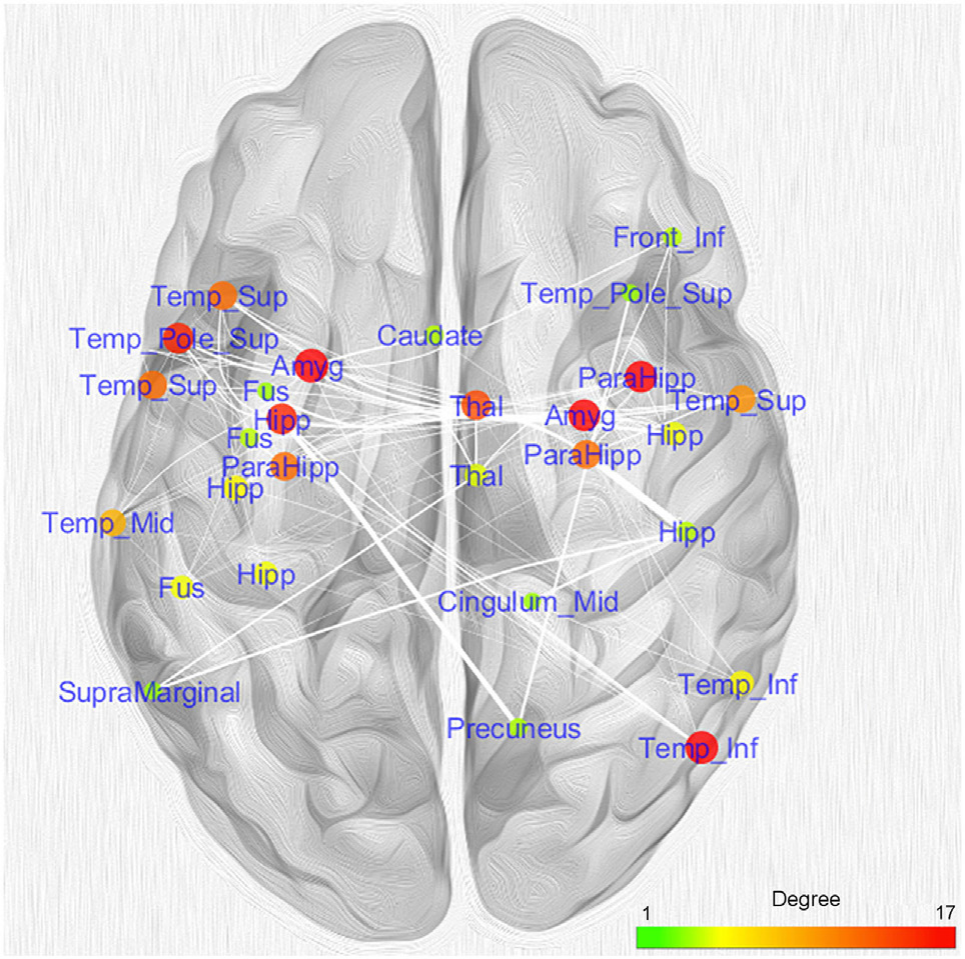
Topological analysis of the coatrophy network of Alzheimer’s disease. Nodes referring to the same brain areas or strictly close one to the other have been collapsed in a single node.

Figure 5 shows the nodes according to their anatomical position. In order to simplify the visual interpretation, we have merged two or more nodes referring to the same brain area; however, we have kept the edges unchanged. It is worth noting that the coatrophy network of AD is composed of more interhemispheric (75) than intrahemispheric edges (71). Apart from the hippocampus, most of the inter-hemispheric connections link structures in the medial temporal lobes. Furthermore, unilateral nodes in the right inferior temporal gyrus and right precuneus are linked to areas of both hemispheres.

**Figure 6:**
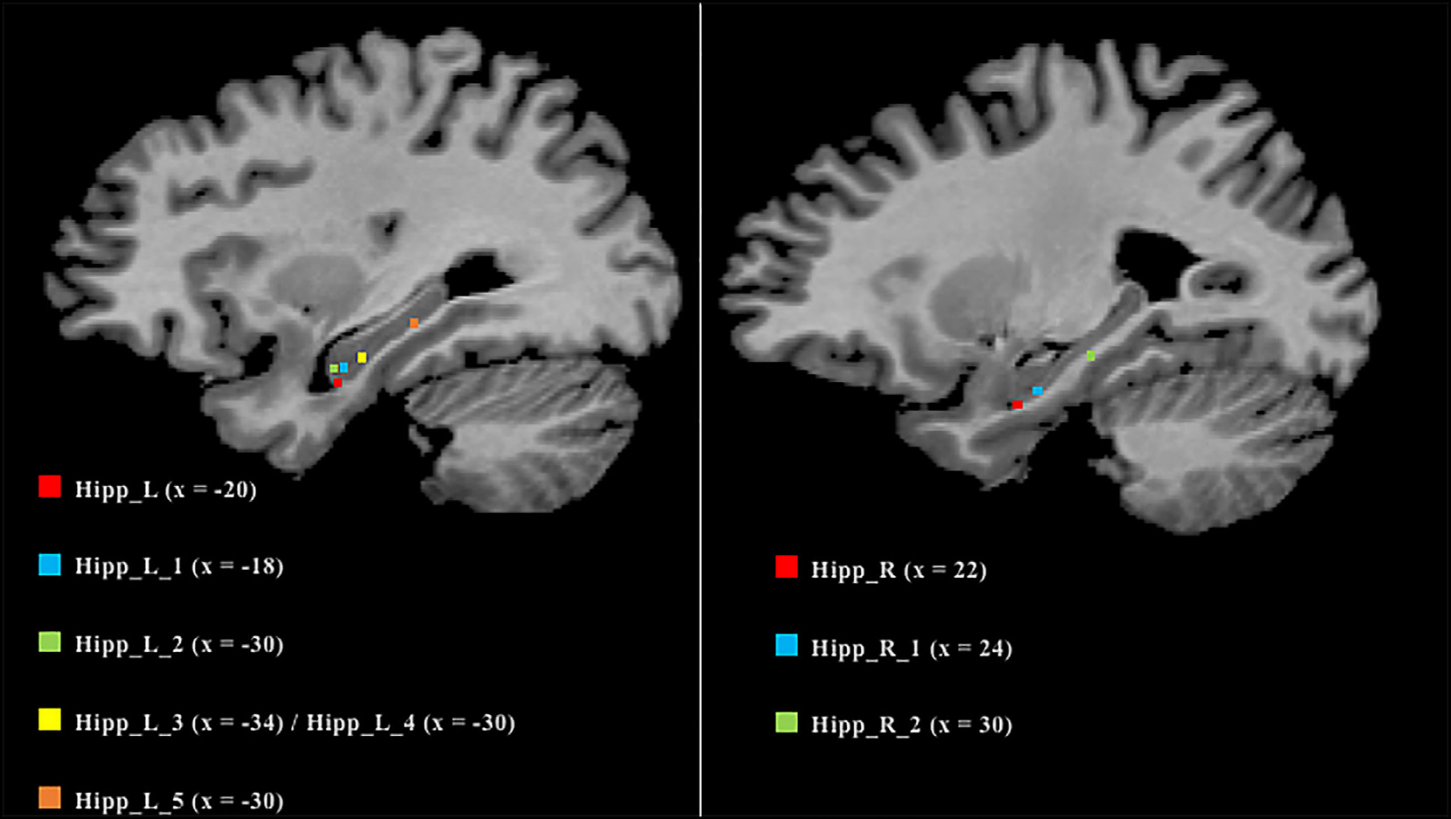
Anatomical localization of the nodes in the hippocampi. Coordinates refers to Talairach space (right sagittal slice *x* = 25, left *x* = 30). Nodes are numerically labeled according to a rostrocaudal criterion.

As many nodes populate the hippocampi, we projected them on a 2D template in order to better clarify their spatial localization (Figure 6). Five out of the six nodes in the left hippocampus were found to be located in the anterior part, while the remaining one was found to be located in the posterior section. In contrast, the right hippocampus exhibits a more uniform pattern, with two anterior nodes and one posterior.

**Figure 7:**
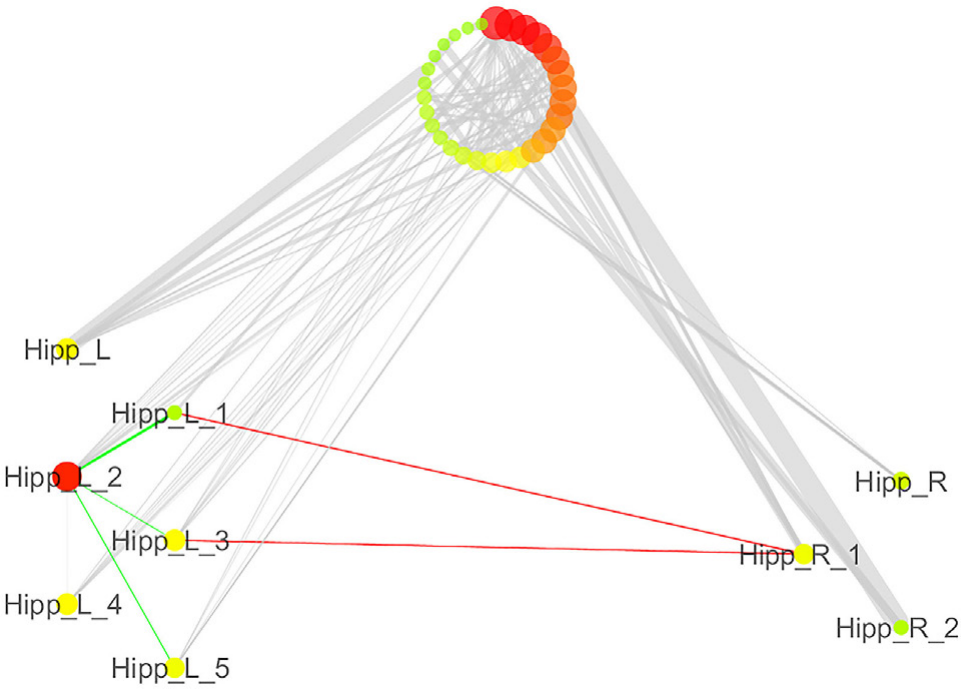
Detailed illustration of the role of the hippocampi in the coatrophy network of Alzheimer’s disease. Green edges are intrahemispheric, while red edges are interhemispheric.

We also analyzed the connectivity profile of the hippocampi within the coatrophy network so as to better understand their relationship with the other nodes of the network (Figure 7). Even though hippocampi have a lot of connections, they are scarcely interconnected (red edges) and, in particular, between the nodes of the right hippocampus there are no direct paths linking them to each other. What is more, the left hippocampus presents a greater number of edges (45) than the right hippocampus (15); however, these edges are generally characterized by a low degree of edge betweenness. In contrast, the 15 edges linking the right hippocampus to the other nodes of the coatrophy network are characterized by a high degree of edge betweenness. Overall, considering the anatomical topology of nodes (Figure 6), the left anterior hippocampus appears to be the most densely connected.

**Figure 8:**
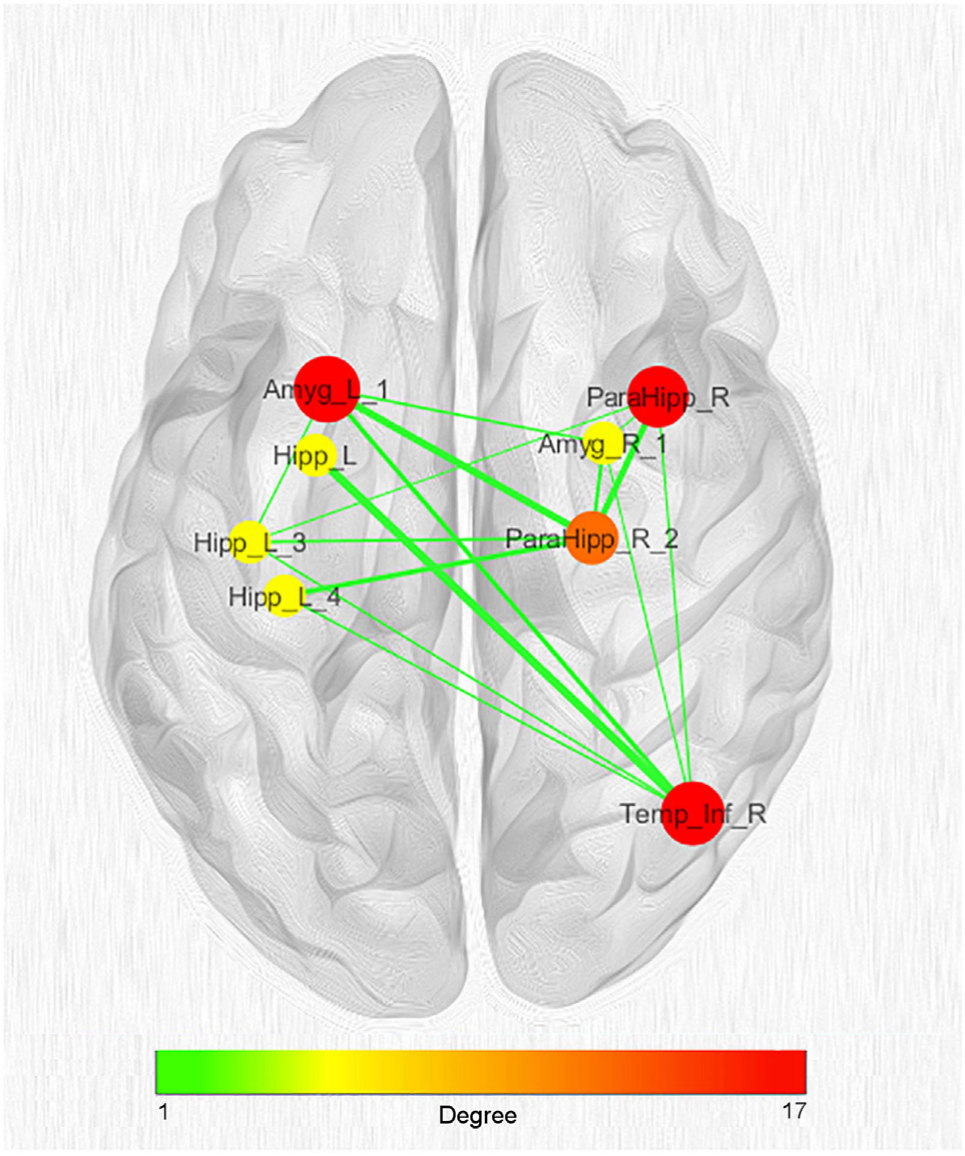
Network clustering with *k*-core decomposition algorithm. Colors and dimensions of nodes indicate their topological degree (smaller node = lower degree; from green to red = from lower to higher values). Thickness of edges indicate the degree of edge betweenness (smaller edge = lower degree). Both node degree and edge betweenness values refer to the original coatrophy network.

Given the great number of nodes and the high density of edges of the coatrophy network, we used the *k*-core algorithm to identify the most connected components of the network. The analysis reported a core subnetwork formed by eight interhemispheric nodes (Figure 8), including the left and right amygdalae, left hippocampus, right parahippocampal gyrus, and right temporal inferior gyrus. The bilateral presence of nodes within this core subnetwork is consistent with the finding that the coatrophy network is characterized by a large number of interhemispheric edges.

## Discussion

With an innovative voxel-based meta-analytic method, this study aimed to find out whether gray matter decreases caused by AD distribute throughout specific and identifiable areas rather than affect randomly the whole brain. After constructing a morphometric coatrophy network, we intended to identify which brain areas are more likely to be altered in conjunction with other ones rather than alone. Finally, we examined the potential existence of relevant subcomponents within the coatrophy network.

The gray matter decreases evaluated by ALE involve limbic and temporal areas, in particular the hippocampus and parahippocampal gyrus. This finding is in accordance with most of previous research (30, 113). Nine out of 40 nodes of the coatrophy network are localized within the hippocampus. Specifically, six nodes are in the left hippocampus (five in its anterior part, one in its posterior part) and three in the right one (two anterior, one posterior). This is consistent with the neuropathological studies suggesting that AD is characterized by an earlier and greater involvement of anatomical structures (including hippocampus) in the left hemisphere (114–116). Although there is still debate about the exact functional organization of the hippocampus (117), the neuroscientific community has achieved a substantial consensus on its role in learning and memory (118), which are both deteriorated cognitive functions in AD. According to Thal et al. (119) the hippocampus (in particular the subfields CA1 and subiculum), along with the amygdala, are pretty soon affected by Aβ plaques during AD evolution (120). In line with AD diagnostic criteria (121) hippocampal and mesial temporal lobe atrophy have been considered as biomarkers of neuronal degeneration, potentially increasing the probability of an underlying AD pathophysiological process. Currently, however, the routinely utilization of hippocampal atrophy in clinical practice is not fully standardized, but preferentially applied in investigational studies and clinical trials. Furthermore, hippocampal atrophy rate could be better accounted for as a sensitive marker of disease progression (122, 123), being able to trace AD natural development and potentially representing an interesting surrogate marker for disease-modifying clinical trials (124, 125). Interestingly, an increased hippocampus and an asymmetry in the shape of the amygdala during the development of AD have been recently demonstrated, with significant correlation to cognitive impairment (126).

According to our analysis, the gray matter coatrophy network of AD appears to be densely interconnected, as suggested by the presence of 146 edges and 40 nodes, 39 of which have at least one connection. The existence of a set of nodes (altered areas) is not a proof *per se* that the disease is spreading. In fact, generally speaking, Patel’s *k* is not always able to identify edges between nodes, which means that, even though some areas are altered, there is no apparent temporal coherence in their capitulation to the disease. The fact, though, that our analysis was able to discover a significant number of edges between nodes is proof of the good reliability of our results pointing out that the alteration cooccurrence really happens, as well as of the consistency of our sample.

Our analysis suggests that AD tends to target a somewhat limited set of brain regions, rather than randomly affecting distinct sites. Furthermore, the left hippocampus, bilateral amygdala, right parahippocampal gyrus, and right inferior temporal lobe seem to follow a very similar pace of degeneration (Figure S4 in Supplementary Material).

In order to evaluate the likelihood of each node of the coatrophy network to be coaltered with other ones rather than as an individual spot we calculated their node degree. The highest value pertains to the node of the left amygdala, which is reached by 17 edges, but we found other 13 nodes with at least 10 edges. These nodes are localized in the temporal lobes, right amygdala, parahippocampal gyrus, left hippocampus, and right thalamus. The high degree of pathoconnectivity of these nodes suggests that, when gray matter alteration affects one of them, it is highly probable that many other regions are also found to be altered. It is also true the other way round, that is, when nodes characterized by low degree show atrophy, it is very likely that this process cooccurs in one of the high-degree nodes, rather than in another low-degree node. These results, as well as the *k*-core decomposition, provide evidence that in the coatrophy network of AD certain nodes have the characteristic of being *pathoconnectivity hubs*. Furthermore, the values of the edge betweenness distribution indicate the existence of a dense subnetwork, which is composed of the nodes with the higher degree of pathoconnectivity.

The paucity of connections linking the two hippocampi suggests a limited cooccurrence of alterations between them. The hippocampus is known to be greatly affected by AD, and the MRI volume estimation of this structure is currently considered one of the most reliable *in vivo* biomarker of this disease (62). Our results suggest that both the hippocampi are substantially altered, albeit somewhat independently. According to previous studies, certain molecular alterations typical of AD are more evident in the left hippocampus compared to the right one (127, 128). This discovery might explain the abundance of edges connecting the nodes in the left hippocampus, as well as support the transneuronal spread mechanism in AD. The nodal stress hypothesis could also play a role in virtue of the intense functional activity of this region. Finally, our finding that the anterior part of the hippocampus exhibits a greater number of edges than the posterior part seems consistent with the suggestion that the deterioration of CA1 and subiculum appears to be more correlated with the development of AD than the deterioration of CA3, which appears to be more correlated with healthy aging (11, 120). Recently, the presubicular–subicular complex has been described as one of the earliest site of atrophy in AD, with a significant correlation with memory performances (even in MCI phase), potentially reflecting the ongoing degenerative process through the subiculum passing from entorhinal cortex to dentate gyrus (129, 130).

In addition to the interpretation of the coatrophy network as a whole, some specific aspects deserve a detailed consideration. The first is the relationship between hippocampus and precuneus. In the coatrophy network of AD these regions are linked through an edge exhibiting a very high degree of edge betweenness, which reveals a direct interaction. According to the “hippocampus disconnection hypothesis” proposed by Tahmasian et al. (131), the disruption of functional connectivity between hippocampus and precuneus could induce the characteristic alterations in the hippocampus that we find in AD. Tahmasian et al. (131) have in fact demonstrated that in AD the hippocampus is much less inhibited, and this disinhibition may result in its hypermetabolism. A similar situation could induce neurotoxicity, which might be one of the causes behind gray matter decrease measured with VBM, thus explaining the identification of a significant number of nodes in the hippocampus.

A second interesting aspect is the relationship between the left hippocampus and right inferior temporal gyrus, which was highlighted by *k*-core decomposition. This result is in agreement with the study of Wang et al. (132), which found that the interaction between these two areas is typical of AD. Of note, Wang et al. (132) examined 80 pathological subjects using Bayesian network analysis and prior-defined regions of interest, while the present study has applied a meta-analytical approach on a substantially bigger VBM database of 883 patients diagnosed with AD. This agreement supports the sensitivity of our novel methodology. Furthermore, the slight prevalence of inter-hemispheric connections in the coatrophy network of AD (see Figure S5 in Supplementary Material) is consistent with the deterioration of white matter bundles in AD, in particular concerning the corpus callosum (133–137). Callosal atrophy has been associated with cognitive decline rate as well as to disease progression (138, 139).

Gray matter alterations found in the hippocampus, precuneus, and inferior parietal cortex can be ascribed to the general disruption of the DMN in patients with AD (58, 140). Recently, a study has showed that the DMN dysfunction, as well as the disruption of the interaction between different resting state functional networks, can be attributed to amyloid burden (58). What is more, Chang et al. (141) have found that amyloid burden in the cingulate cortex might promote gray matter atrophy in the other areas constituting the DMN.

Overall, the crucial role played by pathological proteins in AD supports the transneuronal spread hypothesis at the basis of gray matter alterations’ distribution (4, 5, 39, 40, 42, 45). However, the complex relationship among different factors (such as amyloid burden, Tau deposition, gray matter atrophy, and disrupted functional connectivity) and the presence of several hub nodes within the coatrophy network of AD suggest that the nodal stress mechanism could as well be involved in the development of the disease (142). Therefore, it is extremely likely that different spreading mechanisms, which are not mutually exclusive, may be involved in the etiology of AD.

### Limitations and Future Directions

The present investigation and the methodology on which it is based aim to better understand the nature of AD by examining its pathological fingerprints over the brain. To do so, we were able to get access to a very large sample size of patients. If this is an advantage on the one hand, it can also be a limitation on the other, as within this sample it was not possible to determine the average duration of disease, due to unavailability of information in the original studies. This aspect makes it difficult to associate the coatrophy network with a specific stage of AD progression. However, the methodological procedure for defining the areas to be included in the coatrophy network considers primarily the frequency of every single area to be found altered. In case of a neurodegenerative condition such as Alzheimer’s we could imagine, generally speaking, a group of patients with a recent diagnosis exhibiting alterations in area A, another group with an intermediate development of the disease exhibiting alterations in areas A–B, and another group with an advanced development of the disease exhibiting alterations in areas A–B–C. Since our methodology privileges the frequency of each area to be found altered, in the final network area A will be more likely to be represented, while area C may be even excluded. Moreover, even if the group of patients exhibiting alterations in A–B–C were greater than the other groups, the pattern A–B–C would be less likely to be represented than the sole area A. For this reason, even if our input data could contain an overrepresented sample of patients in a specific stage of the disease, the resulting coalteration network would not represent the pattern of altered areas which is typical of that stage.

Future studies on longitudinal data analyzed by different methods are needed in order to investigate the sequential formation of the coatrophy network identified in this study, so as to achieve a more detailed picture of the temporal evolution of AD.

## Conclusion

This meta-analysis was able to address the following important issues.

- In AD, gray matter alterations do not occur randomly across the brain but, on the contrary, follow identifiable patterns of distribution.
- This alteration pattern exhibits a network-like structure composed of coaltered areas that can be defined as *coatrophy network*.
- Within the *coatrophy network* of AD, certain brain areas, in virtue of their node degree and values of edge betweenness, can be considered as *pathoconnectivity hubs*. The alteration of these areas is supposed to imply a wider distribution of gray matter abnormalities across the brain.
- Within the *coatrophy network* we can identify a core subnetwork of coaltered areas that includes the left hippocampus, left and right amygdalae, right parahippocampal gyrus, and right temporal inferior gyrus.

The innovative methodological analysis developed in this study for constructing the morphometric coatrophy network of an important neurodegenerative disease such as AD opens a new window into the comprehension of the pathological brain. Increasing evidence is supporting the idea that brain alterations distribute according to a network-like structure. The analysis carried out in this study not only provides support for this hypothesis but also puts forward the significant finding that certain nodes of the coatrophy network may play the role of pathoconnectivity hubs. What is more, our methodology can be equally applicable to study the morphometric coalteration network of any other neuropathological condition. Future investigations into this line of research on databases of different diseases promise to provide valuable insight to the study of the dynamics of brain disorders, so as to achieve a better predictive diagnostic power as well as to improve medical care and treatment.

## Author Contributions

JM and AN implemented data collection, analyzed the data, drafted, and revised the article. EP, BB, and KT drafted and revised the article. TC designed the analysis tool, supervised data analysis, drafted, and revised the article. DL retrieved information of the sampled population and implemented bibliographic research. SD revised the article. FC conceived the experiment, supervised data collection, supervised data analysis, drafted, and revised the article.

## Conflict of Interest Statement

The authors declare that the research was conducted in the absence of any commercial or financial relationships that could be construed as a potential conflict of interest. The reviewer AK and handling editor declared their shared affiliation.
