# Supplementary material for: The Pathoconnectivity Profile of Alzheimer’s Disease: A Morphometric Coalteration Network Analysis
Source: Front Neurol. 2018 Jan 25;8:739. doi: 10.3389/fneur.2017.00739 (PMC5810291; doi:10.3389/fneur.2017.00739)
Supplement: Supplementary file 1 [file Data_Sheet_1.docx]

Supplementary Material

The Pathoconnectivity Profile of Alzheimer’s Disease:

A Morphometric Co-alteration Network Analysis

Jordi Manuello^12§^, Andrea Nani^123§^, Enrico Premi^4^, Barbara Borroni^4^, Tommaso Costa^12*^, Karina Tatu^12^, Donato Liloia^2^, Sergio Duca^1^, Franco Cauda^12^

^1^GCS-fMRI, Koelliker Hospital and Department of Psychology, University of Turin, Turin, Italy

^2^ FOCUS Lab, Department of Psychology, University of Turin, Turin, Italy

^3^Michael Trimble Neuropsychiatry Research Group, BSMHFT, Birmingham, UK

^4^ Neurology Unit, Department of Clinical and Experimental Sciences, University of Brescia, Brescia, Italy

^§^These two Authors contributed equally

*** Correspondence:**Tommaso Costa, PhD
GCS fMRI, Koelliker Hospital and Department of Psychology
University of Turin
Via Verdi, 10  10124 Turin (Italy)
phone number: (+39) 011.670.29.80
fax: (+39) 011.814.62.31
e-mail: tommaso.costa@unito.it

# Selection of studies

The BrainMap database was employed for the retrieval of relevant brain imaging experiments carried out with the voxel-based morphometry (VBM) technique. As reported in the User Manual, BrainMap uses a structured standardized coding scheme that describes published human neuroimaging experimental results. This taxonomy has been used to describe over 3600 publications and 15000 experiments, drawing upon over 110000 subjects and reporting over 120000 coordinates. The main division of the coding scheme is between *structural* (VBM) and *functional* data. For this meta-analysis only papers labeled as “Structural” have been used. So, considering only the studies in this category, the database consists of 980 papers, 3093 experiments, 73938 subjects and 21481 locations.

The software application “Sleuth” has been used to search the database for experiments of interest and view the relevant search results in a standard brain space. This procedure allowed us to identify 42 studies about Alzheimer’s disease (AD) for a total of 72 experiments.

Two expert researchers have reviewed all the experiments, so as to ensure: (1) both the presence of the healthy control group and the pathological sample; (2) that results describe differences between subjects diagnosed with AD and healthy controls, and not between AD and other pathologies or between subcategories of AD; (3) that results concern VBM parameters (See Fig. S1 [PRISMA flow chart] and Table 1).

All the selected experiments that did not meet the inclusion criteria were excluded.

Relevant descriptive information was extracted from each article. After this searching procedure, 36 papers were included in the meta-analysis, for a total of 57 experiments and 883 subjects. Table S1 provides a detailed description of the sample of the selected studies.

# Supplementary Figures and Tables

##
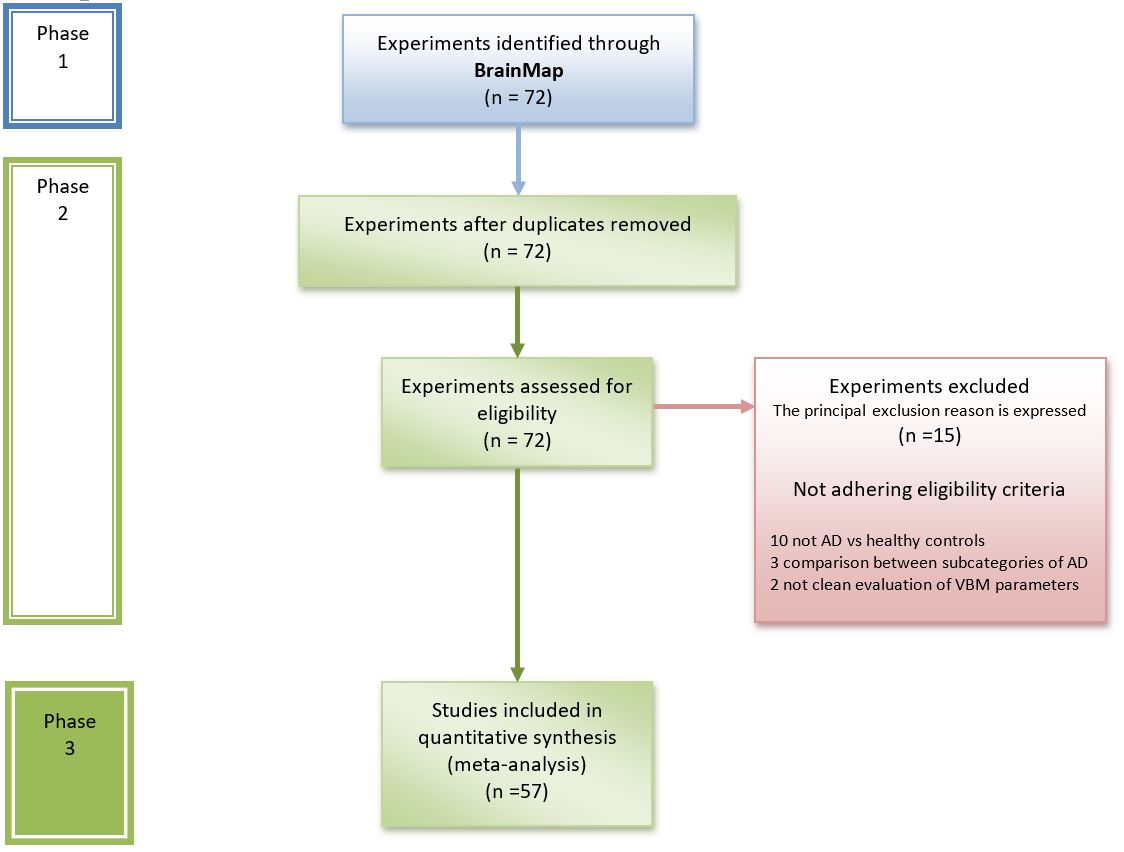
Supplementary Figures

**Figure S1**. Overview of the selection strategy. Numbers refer to experiments.


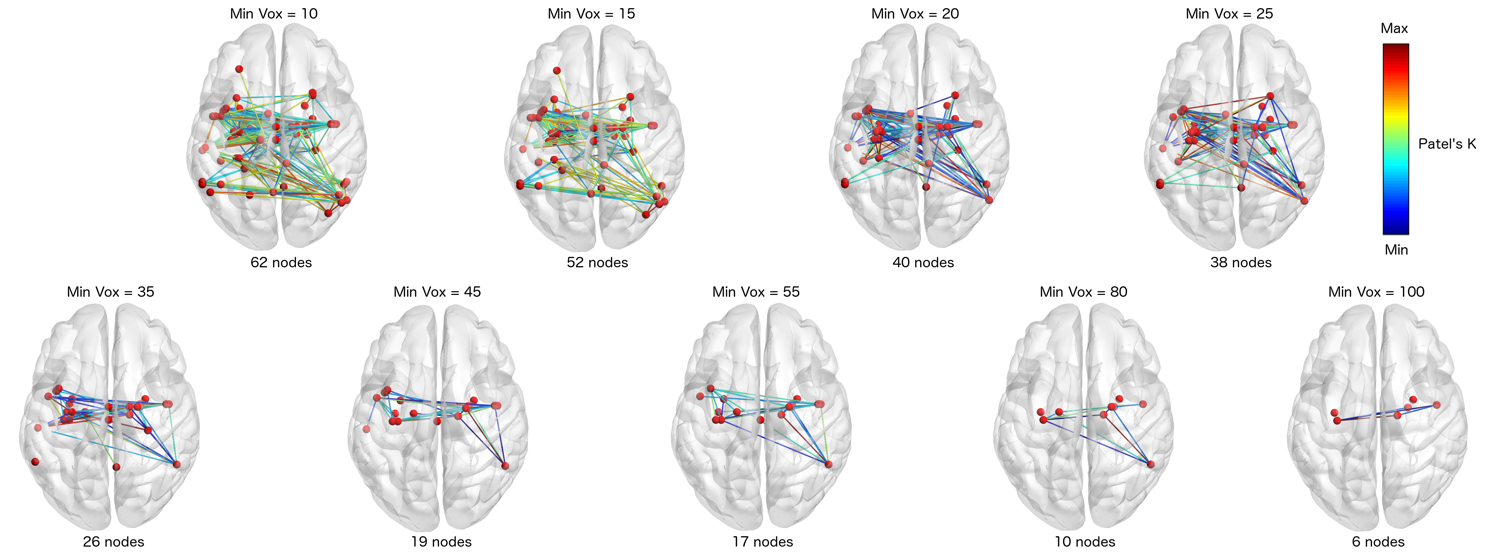


**Figure S2**. The co-alteration network obtained with different values for the minimum number of voxels threshold. Colors from blue to red indicate increasing Patel’s k values (i.e., increasing co-alteration probabilities).


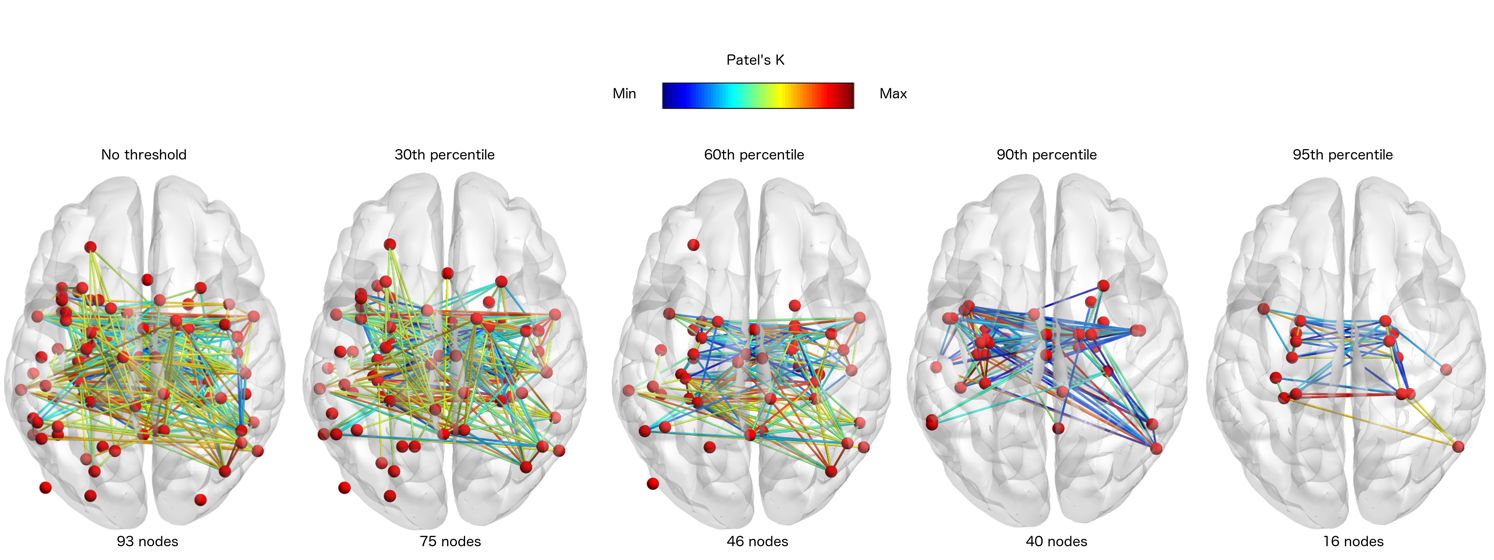


**Figure S3**. The co-alteration network obtained with different values for the peaks’ values percentile threshold. Colors from blue to red indicate increasing Patel’s k values (i.e., increasing co-alteration probabilities).


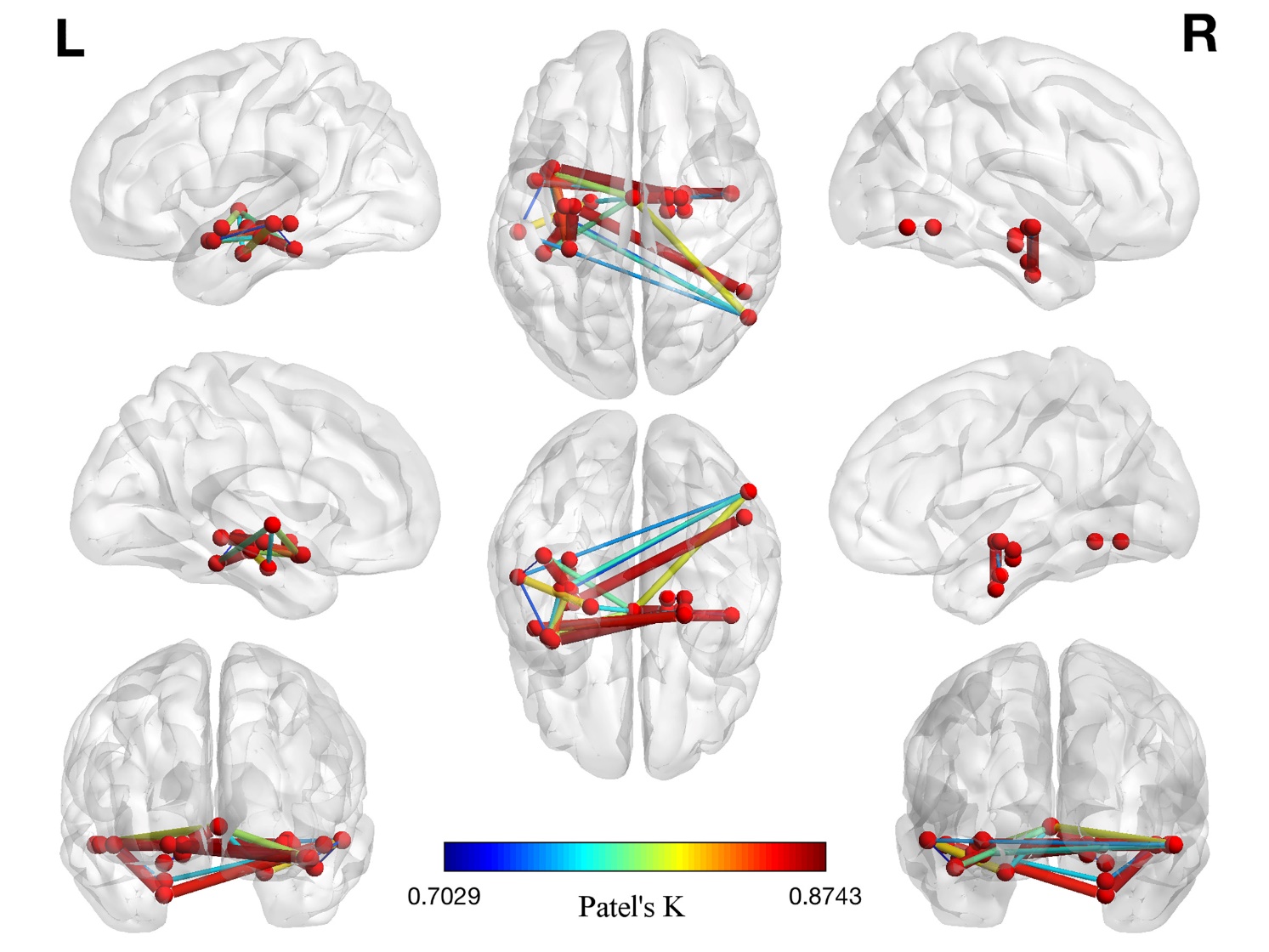


**Figure S4.** Results of the morphometric co-atrophy network of AD. Colors from blue to red indicate increasing Patel’s k values (i.e., increasing co-alteration probabilities). Only edges with k > 0.7 are shown.


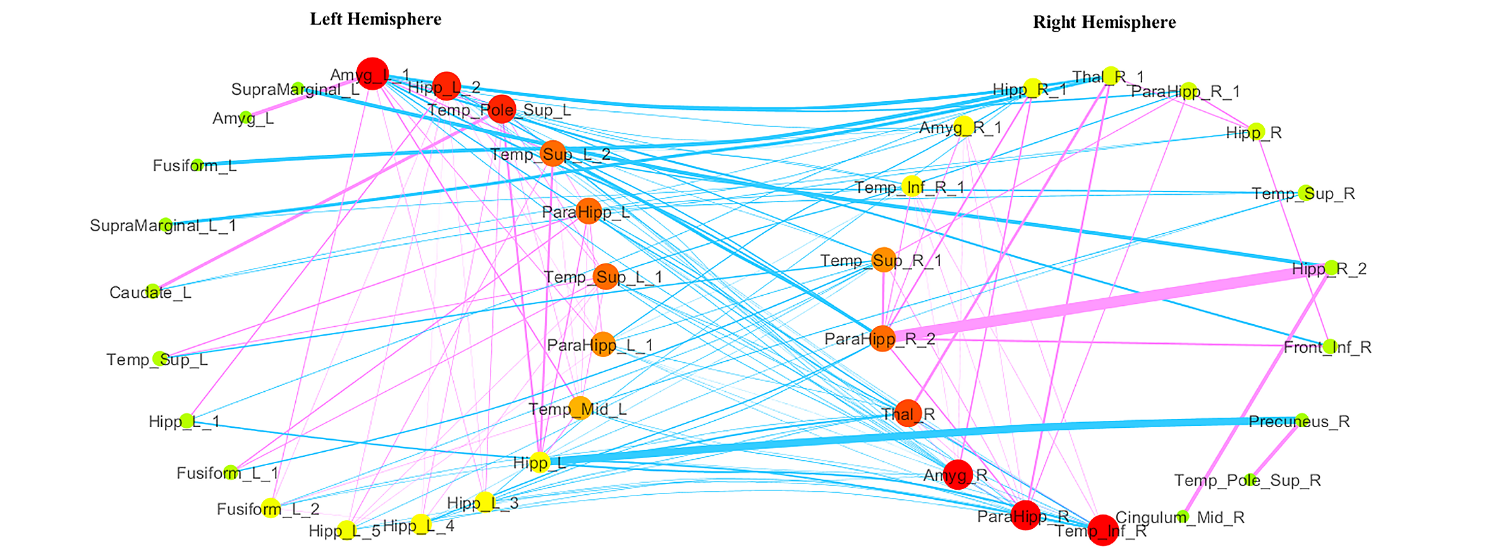


**Figure S5**. Distribution across hemispheres of the co-atrophy network of AD. Violet edges are intra-hemispheric, while blue edges are inter-hemispheric.

**2.2 Supplementary Tables**

| **Node Name** | **Corresponding brain area** | **Node Talairach coordinates** | | | **Node Degree** |
| --- | --- | --- | --- | --- | --- |
|  |  | **X** | **Y** | **Z** |  |
| Amyg_L_1 | Left Amygdala | -22 | -2 | -24 | 17 |
| Temp_Inf_R | Right Inferior Temporal Gyrus | 54 | -64 | -10 | 16 |
| Amyg_R | Right Amygdala | 16 | -8 | -10 | 15 |
| ParaHipp_R | Right Parahippocampal Gyrus | 24 | -6 | -32 | 15 |
| Hipp_L_2 | Left Hippocampus | -30 | -10 | -18 | 14 |
| Temp_Pole_Sup_L | Left Superior Temporal Pole | -40 | 4 | -16 | 14 |
| Thal_R | Right Thalamus | 0 | -8 | -2 | 13 |
| ParaHipp_L | Left Parahippocampal Gyrus | -32 | -12 | -10 | 12 |
| ParaHipp_R_2 | Right Parahippocampal Gyrus | 16 | -14 | -14 | 12 |
| Temp_Sup_L_1 | Left Superior Temporal Gyrus | -46 | 0 | -10 | 12 |
| Temp_Sup_L_2 | Left Superior Temporal Gyrus | -38 | 6 | -16 | 12 |
| ParaHipp_L_1 | Left Parahippocampal Gyrus | -28 | -12 | -10 | 11 |
| Temp_Sup_R_1 | Right Superior Temporal Gyrus | 46 | -6 | -10 | 11 |
| Temp_Mid_L | Left Middle Temporal Gyrus | -54 | -24 | -8 | 10 |
| Amyg_R_1 | Right Amygdala | 22 | -8 | -10 | 8 |
| Hipp_L | Left Hippocampus | -20 | -10 | -22 | 8 |
| Hipp_L_3 | Left Hippocampus | -34 | -18 | -14 | 8 |
| Hipp_L_4 | Left Hippocampus | -30 | -18 | -14 | 8 |
| Temp_Inf_R_1 | Right Inferior Temporal Gyrus | 52 | -52 | -10 | 8 |
| Fusiform_L_2 | Left Fusiform Gyrus | -42 | -34 | -20 | 7 |
| Hipp_L_5 | Left Hippocampus | -30 | -32 | -8 | 7 |
| Hipp_R_1 | Right Hippocampus | 24 | -14 | -18 | 7 |
| Thal_R_1 | Right Thalamus | 0 | -18 | 2 | 6 |
| Hipp_R | Right Hippocampus | 22 | -8 | -22 | 5 |
| ParaHipp_R_1 | Right Parahippocampal Gyrus | 24 | -8 | -26 | 5 |
| Temp_Sup_R | Right Superior Temporal Gyrus | 44 | -6 | -10 | 4 |
| Caudate_L | Left Caudate Nucleus | -6 | 2 | 4 | 3 |
| Front_Inf_R | Right Inferior Frontal Gyrus | 28 | 16 | -10 | 3 |
| Fusiform_L_1 | Left Fusiform Gyrus | -32 | -12 | -32 | 3 |
| Hipp_L_1 | Left Hippocampus | -18 | -12 | -18 | 3 |
| Hipp_R_2 | Right Hippocampus | 30 | -26 | -12 | 3 |
| Temp_Sup_L | Left Superior Temporal Gyrus | -48 | 0 | -10 | 3 |
| Precuneus_R | Right Precuneus | 6 | -54 | 16 | 2 |
| SupraMarginal_L_1 | Left Supramarginal Gyrus | -56 | -50 | 28 | 2 |
| Amyg_L | Left Amygdala | -28 | -2 | -24 | 1 |
| Cingulum_Mid_R | Right Midcingulate cortex | 8 | -36 | 34 | 1 |
| Fusiform_L | Left Fusiform Gyrus | -30 | -6 | -36 | 1 |
| SupraMarginal_L | Left Supramarginal Gyrus | -56 | -52 | 28 | 1 |
| Temp_Pole_Sup_R | Right Superior Temporal Pole | 22 | 8 | -20 | 1 |
| Fusiform_R | Right Fusiform Gyrus | 28 | -2 | -42 | 0 |

**Table S1.** Names, Talairach coordinates and node degree of nodes.

|  | **Node** | **Node** | **Patel's K** |
| --- | --- | --- | --- |
| **1** | ParaHipp_L_1 | Hipp_L_5 | 0.8743 |
| **2** | Temp_Sup_L_2 | Amyg_R_1 | 0.8727 |
| **3** | Temp_Inf_R_1 | ParaHipp_L_1 | 0.8606 |
| **4** | Amyg_R | Thal_R | 0.8602 |
| **5** | ParaHipp_R | Temp_Sup_R_1 | 0.8592 |
| **6** | Fusiform_L_2 | Hipp_L_4 | 0.8574 |
| **7** | ParaHipp_R | Temp_Sup_L_1 | 0.8499 |
| **8** | Temp_Sup_L_2 | Hipp_L_5 | 0.8435 |
| **9** | Hipp_L | Temp_Mid_L | 0.8142 |
| **10** | Temp_Inf_R | Thal_R | 0.8065 |
| **11** | Temp_Pole_Sup_L | Hipp_L_4 | 0.7943 |
| **12** | Temp_Pole_Sup_L | Thal_R | 0.7943 |
| **13** | Fusiform_L_2 | Thal_R | 0.7804 |
| **14** | Hipp_L_3 | Temp_Inf_R | 0.7747 |
| **15** | Hipp_L | Thal_R | 0.7630 |
| **16** | Temp_Pole_Sup_L | Hipp_L_3 | 0.7606 |
| **17** | ParaHipp_R_1 | Temp_Sup_L_1 | 0.7598 |
| **18** | Temp_Inf_R | Temp_Mid_L | 0.7493 |
| **19** | ParaHipp_R_1 | Temp_Sup_R_1 | 0.7483 |
| **20** | Hipp_L_4 | Temp_Inf_R | 0.7359 |
| **21** | Temp_Pole_Sup_L | Temp_Mid_L | 0.7338 |
| **22** | Hipp_R_1 | ParaHipp_R_2 | 0.7267 |
| **23** | Fusiform_L_2 | Temp_Mid_L | 0.7164 |
| **24** | Temp_Sup_L_2 | ParaHipp_L | 0.7029 |
| **25** | Temp_Pole_Sup_L | Amyg_R | 0.6901 |
| **26** | ParaHipp_R_1 | Amyg_L_1 | 0.6778 |
| **27** | Fusiform_L_1 | ParaHipp_L | 0.6771 |
| **28** | Hipp_L_3 | ParaHipp_R_2 | 0.6763 |
| **29** | Hipp_L_2 | Hipp_L_5 | 0.6763 |
| **30** | Amyg_L_1 | Temp_Pole_Sup_L | 0.6476 |
| **31** | ParaHipp_R_1 | Hipp_R | 0.6366 |
| **32** | Temp_Inf_R | Amyg_R | 0.6282 |
| **33** | Amyg_L_1 | Fusiform_L_2 | 0.6274 |
| **34** | Hipp_L_2 | Hipp_L_4 | 0.6238 |
| **35** | Hipp_L_4 | ParaHipp_R_2 | 0.6238 |
| **36** | Hipp_L | Precuneus_R | 0.6221 |
| **37** | ParaHipp_L_1 | Temp_Sup_R_1 | 0.6213 |
| **38** | Thal_R_1 | Caudate_L | 0.6202 |
| **39** | Temp_Sup_L_2 | Amyg_R | 0.6039 |
| **40** | Fusiform_L_1 | Temp_Sup_R_1 | 0.6027 |
| **41** | Hipp_L_2 | Temp_Pole_Sup_L | 0.6023 |
| **42** | ParaHipp_R_2 | Amyg_R_1 | 0.6023 |
| **43** | ParaHipp_R_2 | Hipp_R_2 | 0.6018 |
| **44** | ParaHipp_R_2 | Front_Inf_R | 0.6018 |
| **45** | ParaHipp_R | Temp_Inf_R_1 | 0.6014 |
| **46** | Temp_Sup_L_1 | Hipp_L_5 | 0.6000 |
| **47** | Hipp_R | SupraMarginal_L_1 | 0.5824 |
| **48** | Thal_R_1 | SupraMarginal_L_1 | 0.5824 |
| **49** | ParaHipp_R | Temp_Inf_R | 0.5822 |
| **50** | Amyg_L_1 | Hipp_L_3 | 0.5790 |
| **51** | Fusiform_L_2 | Hipp_L_2 | 0.5782 |
| **52** | Hipp_L_2 | Temp_Sup_R_1 | 0.5777 |
| **53** | Amyg_L | Amyg_L_1 | 0.5774 |
| **54** | ParaHipp_L | Temp_Sup_L | 0.5773 |
| **55** | Fusiform_L_2 | Amyg_R | 0.5761 |
| **56** | Hipp_L_2 | ParaHipp_L_1 | 0.5717 |
| **57** | Amyg_R | Temp_Sup_L_1 | 0.5702 |
| **58** | Hipp_R_2 | SupraMarginal_L | 0.5650 |
| **59** | Hipp_R_2 | Cingulum_Mid_R | 0.5650 |
| **60** | ParaHipp_R | ParaHipp_R_1 | 0.5648 |
| **61** | ParaHipp_R | Thal_R_1 | 0.5648 |
| **62** | ParaHipp_R | Temp_Sup_L_2 | 0.5611 |
| **63** | ParaHipp_R | Amyg_R_1 | 0.5611 |
| **64** | Hipp_L_2 | Amyg_R | 0.5510 |
| **65** | Hipp_L_2 | Temp_Sup_L_1 | 0.5505 |
| **66** | Amyg_L_1 | Hipp_R_1 | 0.5417 |
| **67** | Amyg_L_1 | Temp_Inf_R_1 | 0.5390 |
| **68** | Fusiform_L_1 | Temp_Sup_L_1 | 0.5289 |
| **69** | ParaHipp_R_2 | Temp_Inf_R_1 | 0.5281 |
| **70** | Hipp_L_4 | Temp_Sup_R_1 | 0.5269 |
| **71** | ParaHipp_R | Hipp_L_2 | 0.5260 |
| **72** | ParaHipp_L | Thal_R | 0.5236 |
| **73** | Hipp_L_3 | Thal_R | 0.5224 |
| **74** | Hipp_L_1 | Hipp_L_2 | 0.5195 |
| **75** | Hipp_R_1 | Hipp_L_3 | 0.5188 |
| **76** | Hipp_R_1 | ParaHipp_L_1 | 0.5188 |
| **77** | Amyg_L_1 | Temp_Inf_R | 0.5161 |
| **78** | Hipp_L_2 | ParaHipp_R_2 | 0.5147 |
| **79** | Amyg_R | Amyg_R_1 | 0.5137 |
| **80** | Hipp_L | Temp_Sup_R | 0.5123 |
| **81** | ParaHipp_L | ParaHipp_L_1 | 0.5093 |
| **82** | Amyg_L_1 | Hipp_L_2 | 0.5082 |
| **83** | Amyg_L_1 | ParaHipp_R_2 | 0.5082 |
| **84** | ParaHipp_R | Hipp_L_3 | 0.5077 |
| **85** | ParaHipp_R | ParaHipp_L_1 | 0.5077 |
| **86** | Fusiform_L | Hipp_R_1 | 0.5048 |
| **87** | Hipp_L | Temp_Pole_Sup_L | 0.5029 |
| **88** | Hipp_L | Temp_Sup_L_2 | 0.5029 |
| **89** | Hipp_L_2 | Temp_Inf_R | 0.5029 |
| **90** | Hipp_L_4 | Thal_R | 0.5027 |
| **91** | Hipp_L_4 | Temp_Sup_L_1 | 0.5025 |
| **92** | Temp_Inf_R | Temp_Sup_L_1 | 0.5025 |
| **93** | Temp_Sup_L_1 | Thal_R | 0.5025 |
| **94** | Amyg_L_1 | Thal_R_1 | 0.4994 |
| **95** | ParaHipp_L_1 | Amyg_R | 0.4955 |
| **96** | ParaHipp_L | Temp_Sup_R | 0.4946 |
| **97** | Temp_Sup_L_1 | Temp_Mid_L | 0.4944 |
| **98** | ParaHipp_L | Amyg_R_1 | 0.4941 |
| **99** | Amyg_L_1 | Temp_Sup_L_2 | 0.4907 |
| **100** | Amyg_L_1 | Amyg_R_1 | 0.4907 |
| **101** | Temp_Sup_R_1 | Temp_Sup_L | 0.4906 |
| **102** | Temp_Pole_Sup_L | Temp_Sup_R_1 | 0.4897 |
| **103** | Hipp_R | Front_Inf_R | 0.4859 |
| **104** | Hipp_R_1 | Amyg_R | 0.4856 |
| **105** | Hipp_L_1 | Temp_Inf_R | 0.4756 |
| **106** | Thal_R | Thal_R_1 | 0.4756 |
| **107** | ParaHipp_L | Temp_Mid_L | 0.4755 |
| **108** | Temp_Sup_L_2 | ParaHipp_R_2 | 0.4747 |
| **109** | ParaHipp_R | Amyg_R | 0.4741 |
| **110** | Hipp_L_2 | Hipp_L_3 | 0.4687 |
| **111** | Temp_Sup_L_2 | Temp_Sup_R | 0.4681 |
| **112** | ParaHipp_R_2 | Temp_Sup_R_1 | 0.4681 |
| **113** | Temp_Sup_L_2 | Thal_R | 0.4679 |
| **114** | Fusiform_L_2 | Temp_Sup_R_1 | 0.4645 |
| **115** | Temp_Pole_Sup_L | Temp_Sup_L_1 | 0.4645 |
| **116** | Temp_Pole_Sup_L | Caudate_L | 0.4645 |
| **117** | Hipp_R_1 | Hipp_L_1 | 0.4619 |
| **118** | Temp_Pole_Sup_R | Precuneus_R | 0.4619 |
| **119** | Temp_Sup_R_1 | Hipp_L_5 | 0.4576 |
| **120** | Amyg_L_1 | ParaHipp_L_1 | 0.4550 |
| **121** | Temp_Inf_R_1 | ParaHipp_L | 0.4546 |
| **122** | Hipp_R | Thal_R_1 | 0.4545 |
| **123** | Temp_Sup_L | Temp_Sup_L_1 | 0.4519 |
| **124** | Temp_Pole_Sup_L | Temp_Inf_R_1 | 0.4441 |
| **125** | Temp_Sup_L_2 | Temp_Mid_L | 0.4411 |
| **126** | ParaHipp_R | Temp_Mid_L | 0.4386 |
| **127** | Temp_Inf_R | ParaHipp_L_1 | 0.4315 |
| **128** | Temp_Inf_R | Hipp_L_5 | 0.4315 |
| **129** | ParaHipp_L_1 | Thal_R | 0.4315 |
| **130** | Hipp_R | Caudate_L | 0.4312 |
| **131** | Amyg_L_1 | Amyg_R | 0.4311 |
| **132** | ParaHipp_R | ParaHipp_R_2 | 0.4273 |
| **133** | Temp_Inf_R | ParaHipp_L | 0.4225 |
| **134** | Hipp_L | Temp_Inf_R | 0.4150 |
| **135** | Amyg_R | Hipp_L_5 | 0.4128 |
| **136** | ParaHipp_R | Thal_R | 0.4127 |
| **137** | Hipp_L | ParaHipp_L | 0.4104 |
| **138** | Amyg_L_1 | Front_Inf_R | 0.4053 |
| **139** | Temp_Inf_R_1 | Amyg_R | 0.4038 |
| **140** | Temp_Pole_Sup_L | Temp_Sup_L_2 | 0.4006 |
| **141** | Temp_Pole_Sup_L | Amyg_R_1 | 0.4006 |
| **142** | Amyg_L_1 | Temp_Mid_L | 0.3987 |
| **143** | ParaHipp_L | Amyg_R | 0.3917 |
| **144** | Temp_Inf_R | Temp_Inf_R_1 | 0.3914 |
| **145** | Temp_Inf_R | Amyg_R_1 | 0.3749 |
| **146** | Temp_Sup_R | Temp_Mid_L | 0.3666 |

**Table S2.** Patel’s k values of the edges linking each pair of nodes.
